# The Dangers of Social Media as an Information Source: A Case Report of a Burn After Attempted Breast Tightening Using a PlasmaPen

**DOI:** 10.7759/cureus.72885

**Published:** 2024-11-02

**Authors:** Stephen Goulliart, Giulio Jaber, Claudia Miszewska, Carolina Pollio, Socorro Ortiz

**Affiliations:** 1 Plastic and Reconstructive Surgery, Centre Hospitalier Universitaire (CHU) Brugmann, Bruxelles, BEL

**Keywords:** aesthetic procedure, burn, plasma pen, plastic surgery, social media

## Abstract

Cosmetic procedures are a trending topic on social networks. Many users share their experiences and knowledge but also promote procedures whose effectiveness and validity have not been proven, making them seem effective in the collective imagination through the viral effect they generate.

Here, we describe a second-surface-degree burn all over the breast print caused by a non-standard aesthetic procedure popularized on social networks: the PlasmaPen Fibroblast (Sefton, United Kingdom).

## Introduction

Non-surgical skin tightening procedures account for 4.3% of non-surgical aesthetic interventions. These techniques include cryolipolysis, laser, high-intensity focused electromagnetic field, radiofrequency, and high-intensity focused ultrasound. Their use is recommended for fat reduction or improved skin tightening in cases of mild-to-moderate skin laxity after multiple treatments. These medical devices have become a popular alternative, with their cosmetic use regulated by the FDA in the US and the European Commission in the European Union [1,2].

The Plasmapen Fibroblast (Sefton, United Kingdom) is a device that can be bought online and went viral on social media in 2022, highlighting its low cost, quick application, and suitability for at-home use by customers. It employs a treatment method called plasma sublimation, which induces controlled skin damage in the epidermis by generating an electrical arc. This arc is produced by a radiofrequency generator within a handheld electrosurgical unit that ionizes gas particles in the air. Although superficial and do not affect the dermis, the microtraumas are claimed to stimulate fibroblasts to produce collagen and promote collagen contraction, resulting in a Plasmalift effect.

The FDA has approved the device for the removal and destruction of skin lesions in general dermatological procedures (such as keratosis and xanthelasma) and for use as an electrosurgical cutting and coagulation device. However, it is not approved for aesthetic purposes, as indicated in the FDA medical device classification database [3].

On the other hand, procedures by powered microneedle devices and radiofrequency coagulation devices have a code of devices with aesthetic purposes. These methods have demonstrated their effectiveness in treating skin scars, acne, and facial wrinkles. Additionally, they provide lifting effects, improve skin elasticity, enhance collagen production, and cause volumetric fat changes [4].

On social media, the PlasmaPen Fibroblast is touted for its ability to reduce wrinkles, fine lines, skin laxity, and acne scarring, despite the lack of supporting studies. Hernandez et al. examined this trend and concluded that posts about this device are based on unsubstantiated claims by non-medical professionals, promoting its use in unapproved ways. They also cautioned about potential side effects such as burns, hyperpigmentation, and scarring [5].

Physicians currently employ it for its primary indications, namely the destruction of skin lesions and electrosurgical cutting and coagulation. However, it is still being used by self-proclaimed professionals in non-standard ways, posing a risk to patients’ health. Here, we report the case of a forty-four-year-old woman who used PlasmaPen Fibroblast for mastopexy purposes.

## Case presentation

A 44-year-old woman, non-smoker, without any medical or surgical history, was admitted to the emergency department three days after undergoing a non-surgical mastopexy using a PlasmaPen Fibroblast (ForeverLily®), performed by an esthetician after she saw an advertisement for the procedure on social media.

She had never undergone any cosmetic procedure but was enticed by the before-and-after photos and the attractive price, which was four times lower than that of mastopexy surgery.

**Figure 1:**
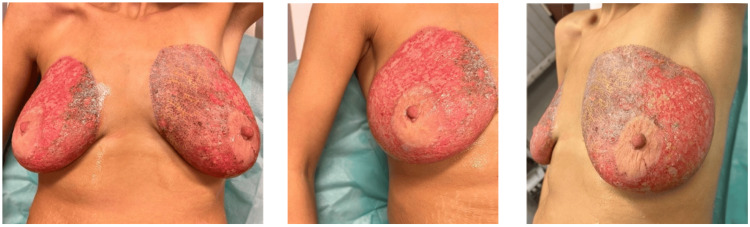
Second-degree burn on the breast print with the NAC complex being spared, three days after undergoing a PlasmaPen Fibroblast procedure. a. front view of breasts, b. right breast, c. left breast

The physical examination revealed blisters, a darker tone with dermal exposure, and a moist appearance involving the skin of both breasts. The diagnosis was a second surface-degree burn all over the treatment area, with the nipple-areola complex being spared (Figure 1).

**Figure 2:**
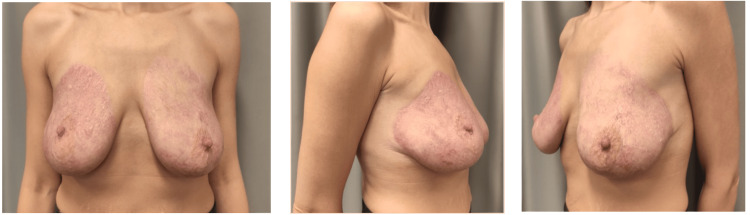
Breast scar 10 days after the burn. a. front view of breasts, b. right breast, c. left breast

Outpatient care was provided: the burn was treated as a thermal injury with an enzyme alginogel (FlaminalÒ) applied every two days. The burn healed in 10 days (Figure 2).

Preventive management of hypertrophic scarring was initiated through conservative therapy, including scar massage, silicone gel, and silicone bandages.

**Figure 3:**
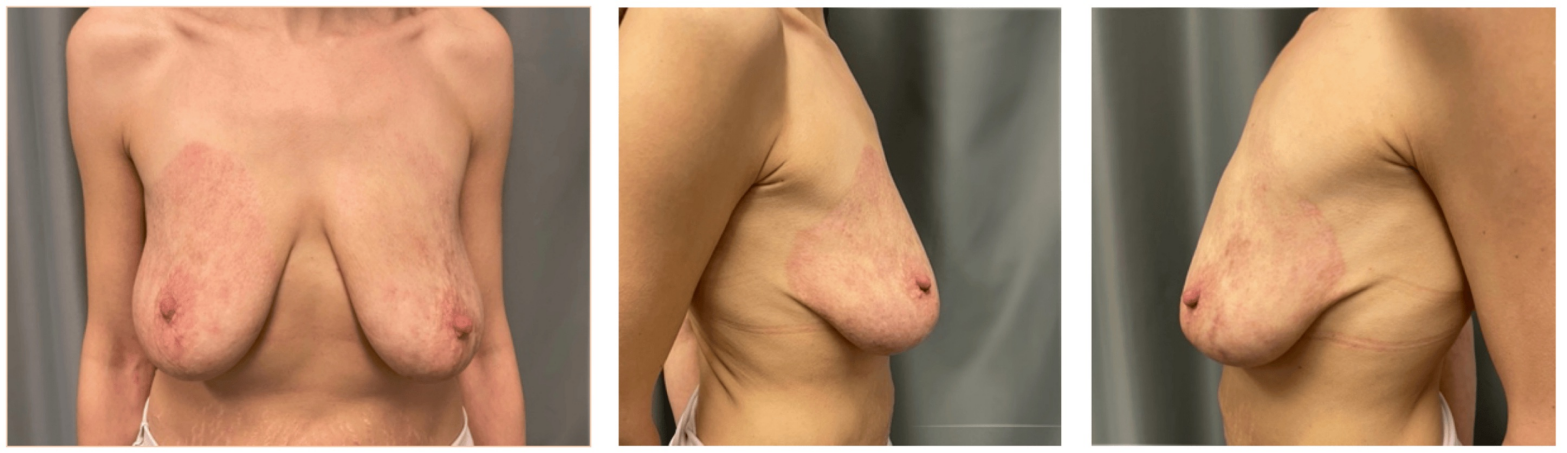
Six-month follow-up showed erythematous and dyschromic scars, especially on the right breast. a. front view of breasts, b. right breast, c. left breast

After six months, the burn resulted in erythematous and dyschromic scars, particularly on the right breast. No hypertrophic or keloid scars were observed (Figure 3).

Neither we nor the patient noted any breast-lifting effect. We recommended a one-year follow-up.

## Discussion

Social media have proven their usefulness for spreading information relevant to public health. However, their use can also lead to risky or inappropriate behavior by users [6-8]. Social media platforms play a central role in how patients perceive themselves and influence the acceptance, normalization, and choice of cosmetic procedures, as well as the intention to undergo them [9-11].

The PlasmaPen Fibroblast is a typical example of this wayward trend: it has gained notoriety through social networking and distribution by non-healthcare professionals. This widespread presence has made it appear as a therapeutic option to patients. Thus, despite the absence of FDA approval for aesthetic use, it is still found and purchased online. This increases the risk of non-standard use by unscrupulous individuals who are unaware of the proper indications and usage, with the primary motive of taking financial advantages from patients.

One approach to addressing these abuses is to regulate how practitioners describe their level of education and qualifications. Transposing this concept to social networks would curb the misleading claims made by non-healthcare professionals about their competencies and help to restore a more professional and ethical practice, especially at a time when the Internet provides limitless outlets for healthcare advertisements. In fact, the American Medical Association (AMA) revealed in their 2020 campaign that patients want all healthcare professionals, physicians, and non-physicians alike, to state their training and licensure clearly [12].

In comparison, many states in the United States have implemented truth-in-advertising laws (TIALs) to limit non-board-certified plastic surgeons from performing cosmetic surgeries beyond their qualifications. However, no statistically significant difference has been found between states with and without TIALs regarding the number of providers who are advertising cosmetic surgeons on social media and who are practicing out of scope [13,14]. This underscores the challenge of regulating aesthetic practices.

Plastic surgeons must be aware of these non-surgical procedure trends that patients are exposed to online. They must educate patients to avoid hype surrounding new devices whose effectiveness and validity have not been proven. Indeed, the American Society of Plastic Surgeons has already highlighted the importance of using social networks for patient education to prevent them from falling prey to misinformation [15].

## Conclusions

Social networks play an increasingly important role in patients’ therapeutic choices. The quest for innovative, more conservative, less invasive, and scar-free techniques exposes patients to misinformation from non-medical professionals.

The call for legislation and awareness campaigns by plastic surgeons on social networks are some recommendations. These measures would help limit the emergence of dangerous and viral procedures, as well as their long-term consequences on patients’ physical and mental health.
